# Dioxygen Reduction at a Cu(I) Complex Supported by a Macrocyclic N_3_O Ligand System

**DOI:** 10.1002/chem.71479

**Published:** 2026-08-14

**Authors:** Maximilian Schütze, Dibya Jyoti Barman, Christian Lorent, Kallol Ray

**Affiliations:** ^1^ Humboldt‐Universität Zu Berlin Institut Für Chemie Berlin Germany; ^2^ Technische Universität Institut Für Chemie Fakultät II Berlin Germany

**Keywords:** bioinorganic chemistry, Cu(I) catalyst, end‐on peroxo, ORR, spectroscopy

## Abstract

Cu^I^(14 TMCO) (**1**), involving a N_3_O‐macrocycle, reacts with O_2_ at cryogenic temperatures to yield an *end‐on* peroxodicopper(II) species. The N_4_‐analogue Cu^I^(14‐TMC) (**2**) remains unreactive under similar conditions. **2** contains an average short Cu─N bond length, resulting in a dominating *trans*‐effect, which inhibits O_2_ binding. In contrast, an elongated Cu─O_TMCO_ bond *trans* to the O_2_‐binding site in **1** allows O_2_‐coordination. The catalytic reduction of O_2_ to H_2_O undergoes a full 4e^−^/4H^+^ process in **1**. Notably, [Cu^I^(12‐TMC)]^+^ (**3**), involving a smaller N_4_‐macrocycle where the Cu(I) ion is forced to move out of the plane formed by the four nitrogen atoms, also binds O_2_. However, in contrast to **1**, **3** mediates the catalytic 2e^−^/2H^+^ reduction of O_2_ to H_2_O_2_ via two ligand‐assisted protonation steps. In this case, the reduction of O_2_ involves the initial formation of a *side‐on* peroxodicopper(II) species, where the weak Cu─O_peroxo_ bond distance resulting from the strong σ‐donation from the *trans* Cu─N bond presumably leads to the release of H_2_O_2_ upon protonation. The present study provides deep mechanistic insights into the transition metal‐mediated O_2_ reduction process and reveals how the first coordination sphere can be tuned to control the 2e^−^/2H^+^ versus 4e^−^/4H^+^ reductions of O_2_.

## Graphical abstract

A non‐reactive [Cu^I^(14‐TMC)]^+^ complex is transformed to an efficient catalyst [Cu^I^(14‐TMCO)]^+^ (TMCO = 4,8,12‐trimethyl‐1‐oxa‐4,8,12‐triazacyclotetradecane) for 4e^−^/4H^+^ reduction of O_2_ by N → O substitution.

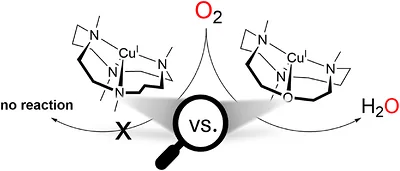

## 1 Introduction

Over the past decades, the activation and reduction of dioxygen (O_2_) by copper complexes have been extensively studied in bioinorganic chemistry, driven by its relevance to enzymatic processes and catalytic oxidation reactions [1, 2, 3, 4, 5, 6, 7, 8, 9, 10, 11, 12, 13, 14, 15, 16, 17, 18, 19, 20, 21, 22]. Several studies have demonstrated that Cu(I) complexes readily react with O_2_ to form a variety of mono‐ and dinuclear Cu/O_2_ adducts such as superoxo, peroxo, and bis‐*μ*‑oxo species, with structures and reactivity patterns that strongly depend on the ligand environment [23, 24, 25]. Systematic modifications of the ligand framework have enabled fine‐tuning of the electronic and steric properties of the metal center, thereby altering O_2_ binding modes, redox potentials, and subsequent reactivity pathways [26, 27].

Among the most extensively studied systems are the polydentate N‐based ligands such as tris(2‑pyridylmethyl)amine (TMPA) and its derivatives, which have yielded a diverse selection of well‑characterized copper–dioxygen intermediates [28, 29, 30, 31, 32]. In parallel, macrocyclic tetradentate ligands such as 14‑ and 12‑membered tetraazamacrocycles like 1,4,8,11‐tetramethyl‐1,4,8,11‐tetraazacyclotetradecane (14‑TMC), 1,4,7,10‐tetramethyl‐1,4,7,10‐tetraazacyclododecane (12‑TMC), and 1,4,8,11‐tetraazacyclotetradecane (cyclam) as well as their N‐ and C‐functionalized analogues, for example, *rac*‐5,5,7,12,12,14‐hexamethyl‐1,4,8,11‐tetraazacyclotetradecane (tet b) have provided additional insight into how macrocyclic ring size and flexibility may influence O_2_ chemistry across the first‑row transition metals (Figure 1) [33, 34, 35]. Notably, while the Cu(I) complexes supported by 14‑TMC are unreactive toward O_2_, the contracted 12‑TMC analogue or the C‐substituted tet b ligand readily bind and activate O_2_ to form *side‑on* and *end‐on* peroxodicopper(II) species, respectively [33, 35].

**FIGURE 1:**
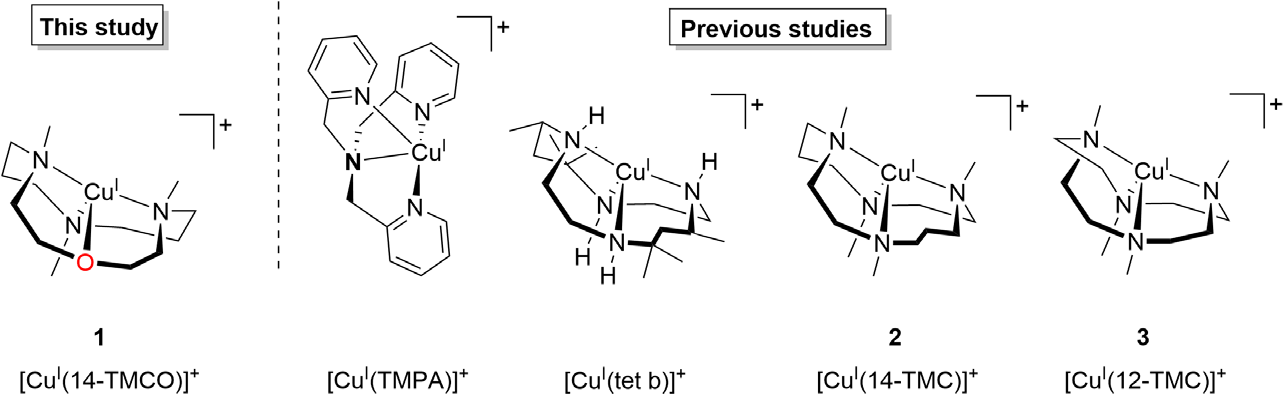
Left: Cu^I^ complex (**1**) employed in this study. Right: Structures of the previously studied Cu^I^ complexes [33, 36].

Despite this progress, most ligand frameworks explored in copper–dioxygen chemistry rely exclusively on nitrogen donors, in spite of the predominance of oxygen‐based ligands in biological systems. In particular, N versus O substitution in iron systems has revealed the profound consequences in the electronic structure and reactivity of the iron‐oxygen intermediates. For example, in a previous study, we demonstrated that replacing one amine donor in the macrocyclic Fe^II^(14‐TMC) complex by an O donor, giving Fe^II^(14‐TMCO) (14‐TMCO = 4,8,12‐trimethyl‐1‐oxa‐4,8,12‐triazacyclotetradecane), drastically enhances O‑atom transfer reactivity and C–H bond activation of substrates through stabilization of a more electrophilic Fe^IV^ = O intermediate [34]. These findings highlight that even a single N → O replacement can significantly influence redox behavior, M–O bond covalency, and reactivity of biologically relevant metal‐oxygen intermediates.

Inspired by these insights, we now explore the effect of analogous ligand modification in copper systems. Herein, we report a comparative study of catalytic O_2_ activation by Cu(I) complexes supported by N_4_‑type (14‑TMC and 12‐TMC) and N_3_O‑type (14‑TMCO) macrocyclic ligands (Figure 1). Notably, in contrast to the unreactive Cu^I^(14‑TMC) complex, the Cu^I^(14‑TMCO) analogue performs a catalytic 4e^−^/4H^+^ reduction of O_2_ to H_2_O. Our results reveal that this single‐donor substitution fundamentally alters the electronic structure of the copper center due to a weakened *trans*‐effect, unlocking previously inaccessible O_2_ activation pathways and highlighting the critical importance of first‑sphere donor tuning in controlling copper–dioxygen chemistry.

## 2 Results and Discussion

### 2.1 Synthesis, Characterization and O_2_ Reactivity of **1**

[Cu^I^(14‐TMCO](OTf) (**1**, OTf = triflate anion, trifluoromethansulfonate) was synthesized via metalation of the 14‐TMCO ligand with Cu^I^(MeCN)_4_OTf in acetone. Electrospray ionization mass spectrometric (ESI‐MS) measurements of **1** showed a peak at *m/z* = 306.2 matching the calculated value and isotope pattern of the ion fragment [Cu^I^+L]^+^, which confirms the successful formation of **1** (Figure S1). The purity of **1** was confirmed by elemental analysis (EA). Diethyl ether diffusion into a solution of **1** in acetone yielded colorless needle crystals suitable for single‐crystal x‐ray diffraction (sc‐XRD) measurements (Figure 2A and Table S1) [37].

**FIGURE 2:**
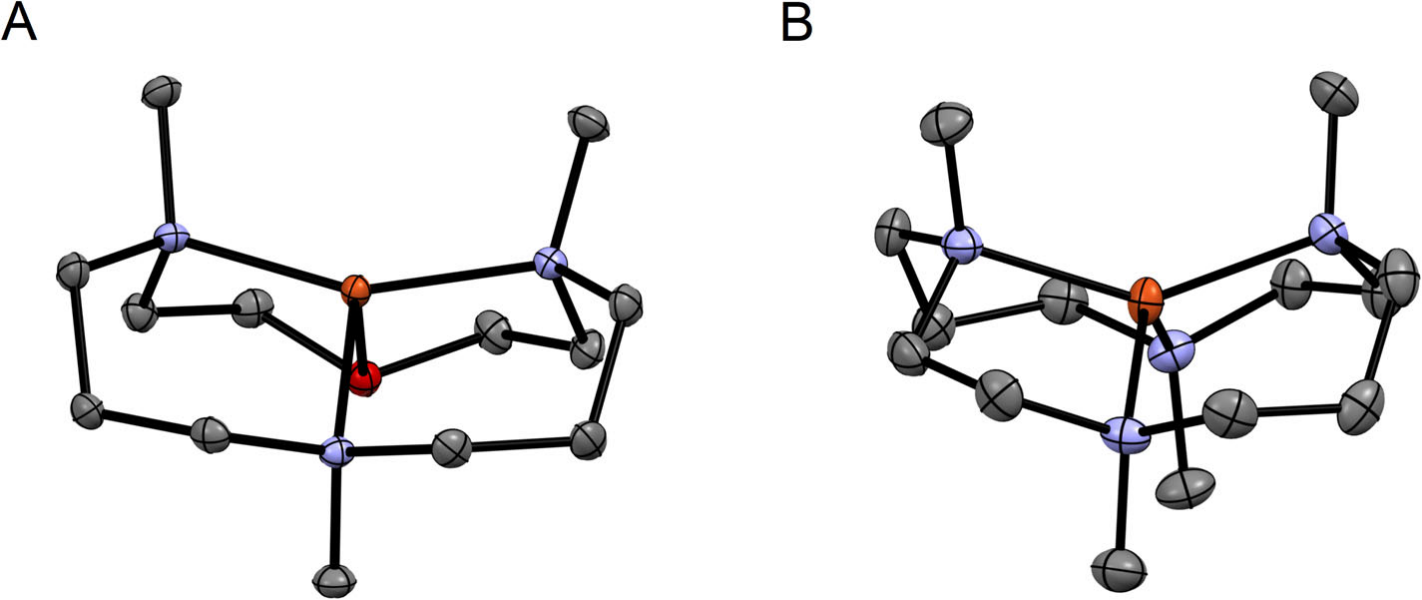
Molecular structure of (A) **1** and (B) **2** obtained by sc‐XRD measurements shown in ORTEP representation with thermal ellipsoids at 50% probability level. H‐atoms and OTf anions are omitted for clarity. (Color code: C: gray, N: blue, O: red, Cu: orange) [38].

Notably, in contrast to the previously reported [Fe^II^(14‐TMCO)(OTf)(CH_3_CN)]^+^ complex, which is 6‐coordinate with axial OTf and CH_3_CN ligands [34], **1** exhibits a 4‐coordinate seesaw geometry at the Cu ion, where the OTf counterion is not bound to the Cu center. A trend of two short (@ 1.985(1) Å), one long (@ 2.081(1) Å) Cu─N and a significantly longer Cu─O (@ 2.360(1) Å) bond length is evident. Interestingly, the two methyl groups are showing in one direction, whereas the third methyl group points in the opposite direction, which again differs from the corresponding iron complexes, where all three methyl groups point in the same direction. For comparison, the N_4_ analogue, [Cu^I^(14‐TMC)](OTf) (**2**), was also synthesized similarly to **1** and analyzed by ESI‐MS (Figure S2) and sc‐XRD measurements (Table S2). Similar to **1**, **2** exhibits a 4‐coordinate seesaw geometry at the Cu ion, where the OTf counterion is not bound to the Cu center (Figure 2B) [38]. In the case of **2**, the two methyl groups are showing in one direction, whereas the other two methyl groups point in the opposite direction (*trans*‐V configuration) [38, 39]. A similar trend of two short (@ 2.034(3) Å and 2.041(3) Å) and two long (@ 2.135(2) Å and 2.121(3) Å) Cu─N bond lengths are evident. The average Cu–N/O bond distances in **1** (@ 2.103 Å) and **2** (@ 2.083 Å) are comparable, suggesting similar electrophilicity of the Cu centers in both cases. Cyclic voltammetric (CV) measurements for **1** and **2** show *E*_Cu_^I/II^ of −0.27 V and −0.37 V versus Fc^+/o^, respectively (Figure S3), consistent with a more electron‐rich Cu center in **2**.

Addition of O_2_ into a solution of **1** in acetone or CH_2_Cl_2_ at −90°C yielded a metastable intermediate (**1a**) with optical features at 527 nm (*ε*_max_ = 3160 M^−1^ cm^−1^) and 888 nm (*ε*_max_ = 740 M^−1^ cm^−1^) (Figure 3A). The binding of O_2_ is thermally reversible, as a fast increase in temperature of the solution initiates the decrease of the optical features corresponding to **1a,** while subsequent cooling of the solution leads to the reformation of **1a**. Additionally, bubbling of Ar through the acetone solution of **1a** for 3 min at −90°C regenerates **1**, further confirming that **1a** is a reversible copper dioxygen adduct; several cycles of alternating Ar/O_2_ purges can be achieved (Figure S4). A slow decrease in the intensity at 527 nm after each cycle can be observed as the reversible binding of O_2_ is competing with a decay process (see below). In comparison, **2** is unreactive towards O_2_ under similar conditions (−90°C in acetone), although its Cu center is more electron‐rich relative to that in **1**.

**FIGURE 3:**
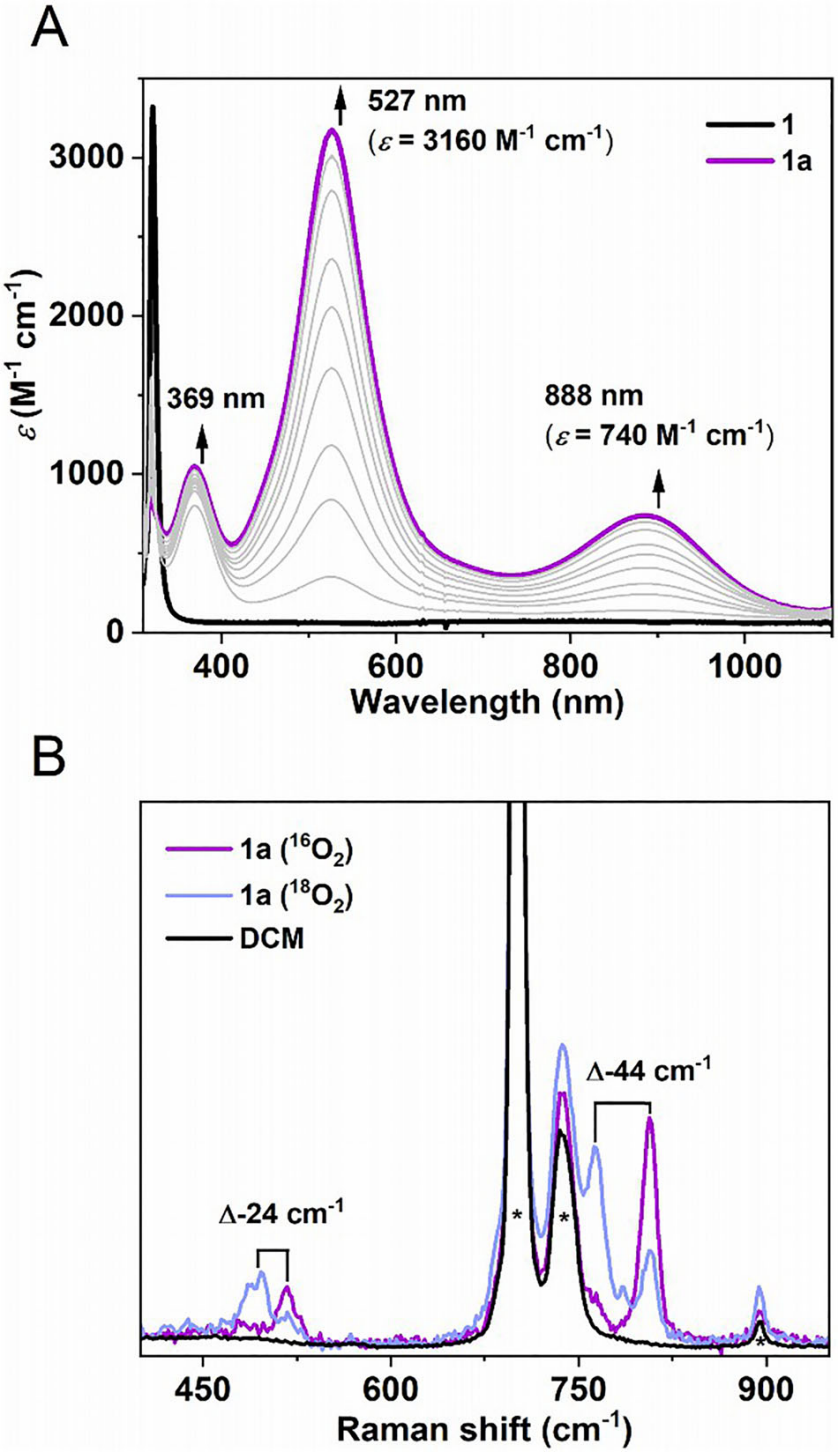
(A) UV/Vis spectral changes associated with the reaction of **1** with O_2_ to form **1a** (temperature: ‐90°C, solvent: acetone); (B) rRaman spectrum of ^16^O_2_‐enriched **1a** (violet), ^18^O_2_‐enriched **1a** (blue) and CH_2_Cl_2_ (black).

Resonance Raman (rRaman) measurements on a CH_2_Cl_2_ solution of **1a** at −95°C upon 532 nm excitation revealed two vibrational modes at 516 cm^−1^ and 807 cm^−1^, which shifted to 492 cm^−1^ (^16/18^Δ = −24 cm^−1^) and 763 cm^−1 ^(^16/18^Δ = −44 cm^−1^), respectively, upon ^18^O isotope labelling. The observed frequencies of these two vibrations are in line with the values expected for the Cu–O and O–O vibrations of a dinuclear *end‐on* Cu‐peroxo species (Figures 3B and S5) [6, 12, 14, 28, 40, 41, 42, 43, 44]. In particular, the O–O vibration in **1a** is comparable to the reported O–O vibrations (811 cm^−1^ and 828 cm^−1^) in the previously reported [35] related [(tet b)CuO_2_Cu(tet, 39b)](OTf)_2_ complex. Additionally, X‐band electron paramagnetic resonance (EPR) measurements on **1a** showed only 5% of an EPR‐active Cu(II)‐based species, suggesting the major species to be EPR‐silent, which is in accordance with typical mono‐ and dinuclear Cu(II)‐peroxo species (Figure S6).

Structural insights were gained from sc‐XRD measurements. Crystals of **1a** were grown via diethyl ether diffusion into a saturated solution of **1a** in acetone at −80°C. **1a** displays a dinuclear structure, where both Cu centers show a distorted trigonal‐bipyramidal geometry (Figure 4), where the O_TMCO_ atom occupies one of the axial positions. The Cu centers are connected via an *end‐on* peroxo moiety, which is *trans* to the O atom of the TMCO ligand. The Cu─O_peroxo_, O─O and the Cu⋯Cu bond distances are determined to be 1.910(2) Å, 1.444(2) Å and 4.6817(5) Å, respectively (Table S3) [37]. In comparison to previously reported *end‐on* peroxodicopper(II) structures, the Cu⋯Cu bond distance in **1a** is significantly more elongated, thereby suggesting a weak binding of O_2_ (Table S4), consistent with the observed reversible O_2_ binding [33, 35, 36, 37]. It is important to note that in **1** the binding of O_2_ occurs at a site *trans* to the oxygen donor of the TMCO ligand; the weak *trans*‐effect exhibited by the long Cu─O_TMCO_ bond presumably provides stability to the otherwise metastable **1a** at cryogenic temperatures. In contrast, all the Cu–N distances in **2** are significantly shorter than the Cu–O_TMCO_ distance of 2.360(1) Å in **1**; correspondingly, the binding of O_2_ to the Cu center suffers from a strong *trans*‐effect, which makes the corresponding Cu‐O_2_‐Cu complex inaccessible, thereby explaining the inability of **2** to activate O_2_.

**FIGURE 4:**
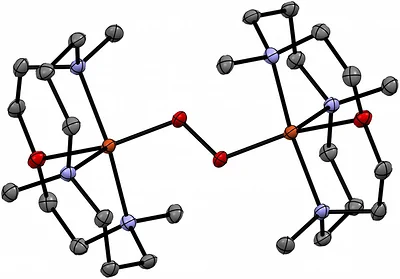
Molecular structure of **1a** obtained by sc‐XRD measurements in ORTEP representation with thermal ellipsoids at 50% probability level. H‐atoms, OTf anions and acetone molecules are omitted for clarity. (Color code: C: gray, N: blue, O: red, Cu: orange) [38].

**1a** is unstable at −80°C in acetone, and spontaneously decays to **1b** with optical features at 369 nm (*ε*_max_ = 1660 M^−1^ cm^−1^) and 723 nm (*ε*_max_ = 316 M^−1^ cm^−1^; Figure 5A). X‐band EPR measurements of **1b** reveal 8% of an EPR‐active Cu(II)‐based species, suggesting the major species is EPR‐silent, which may suggest the formation of a variety of different Cu–O species such as dinuclear Cu(II) hydroperoxo, *side‐on* peroxo, or bis‐*μ*‐hydroxo species (Figure S6). Notably, the O_2_ reactivity of **1** is found to be independent of the anion used; similar formation of **1a** and **1b** was observed in the presence of a non‐coordinating hexafluoro phosphate (PF_6_) counterion (Figures S7–S9 and Table S5). **1b** can also be formed upon addition of 2 eq. of TEMPO‐H (Figure S10) to **1a**. We, therefore, tentatively assign **1b** as a bis(*μ*‐hydroxo)dicopper(II) species. All our efforts to characterize **1b** by ESI‐MS or rRaman spectroscopy, however, were unsuccessful.

**FIGURE 5:**
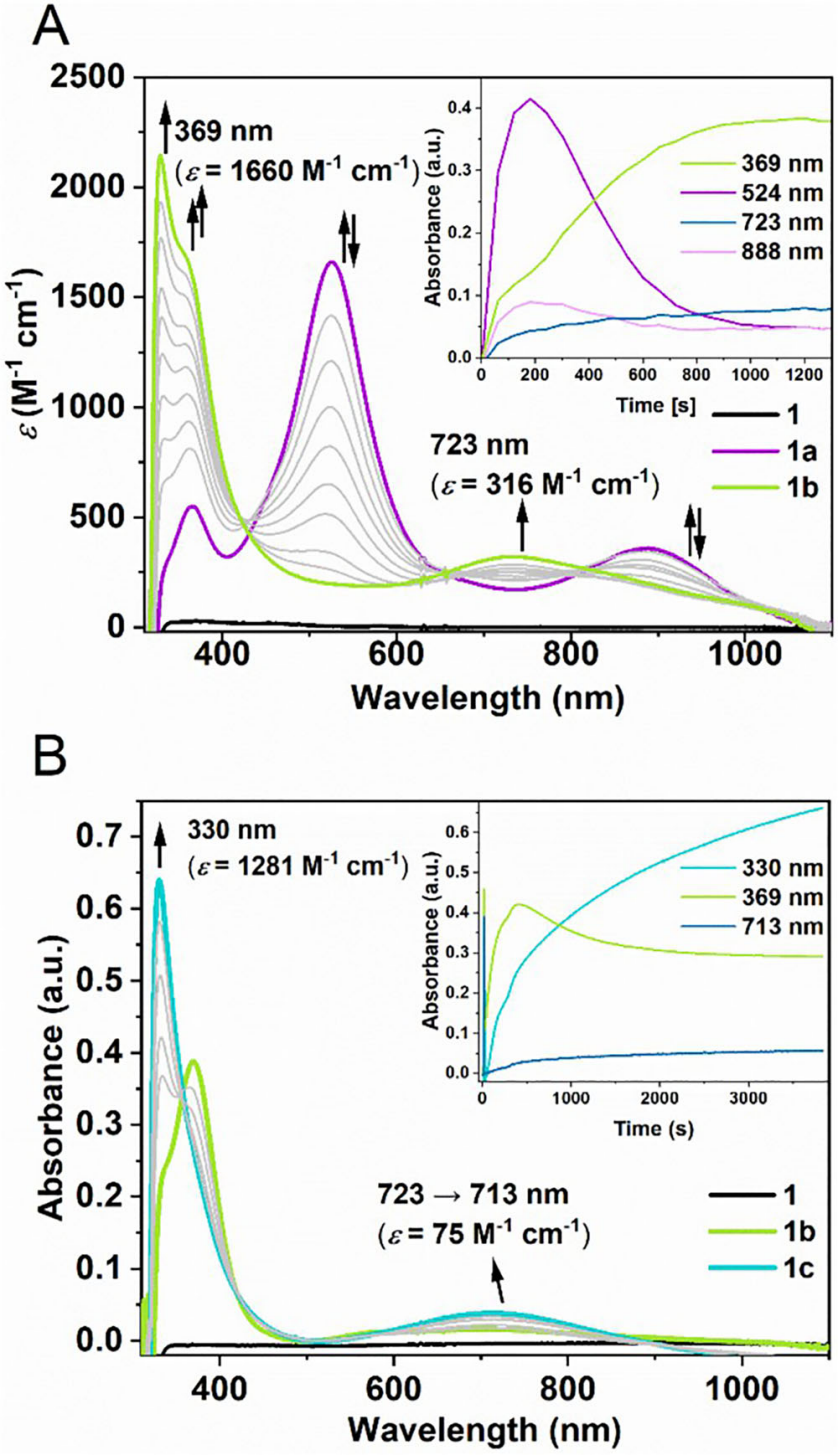
(A) UV/Vis spectral changes associated with the reaction of **1** with O_2_ to form **1a** and its subsequent interconversion to **1b** (solvent: acetone, temperature: −80°C); Inset: Time traces of the bands at 369, 524, 723, and 888 nm. (B) UV/Vis spectral changes associated with the conversion of **1b** to **1c** (solvent: acetone, temperature: −40°C); Inset: Time traces of the bands at 330, 369, and 713 nm.

When the temperature is raised to −40°C, the optical features of **1b** decrease, and the final decay product (**1c**) with optical features at 330 nm (*ε*_max_ = 1281 M^−1^ cm^−1^) and 713 nm (*ε*_max_ = 75 M^−1^ cm^−1^) can be observed (Figure 5B). The structure of **1c**, as obtained from sc‐XRD measurements, shows a square‐pyramidal geometry, where one triflate counterion is bound in an axial position (Figure S11) [38]. All methyl groups are *trans* to the bound triflate ion, suggesting a significant geometrical change upon oxidation of **1** to **1c**. The Cu─N and Cu─O bond lengths are found to be 2.040(1)/2.035(1)/2.031(1) Å and 1.986(1) Å, respectively (Table S6). Notably, the bond lengths in **1c** are more evenly distributed compared to **1**, where the Cu─O_TMCO_ bond was found to be significantly elongated in comparison to the Cu–N bond lengths. Also, the average Cu─N and Cu─O bond lengths (@ 2.023 Å) in **1c** are shorter than those in **1** and **1a**.

### 2.2 Catalytic Dioxygen Reduction Reaction

The evaluation of the catalytic activity of **1** towards O_2_ reduction was carried out using Fukuzumi and Guilard's method [45]; decamethylferrocene (Fc*) was employed as a one‐electron donor, trifluoroacetic acid (TFA) was used as a proton source, and, in their presence, O_2_ was set to react with a catalytic amount of **1** in acetone. The occurrence of the dioxygen reduction reaction (ORR) was proved by the formation of decamethylferrocenium ion (Fc*^+^) with a characteristic absorption band at 780 nm (Figure 6; *ε*_780 nm_ = 520 M^−1^ cm^−1^) [46]. The concentration of Fc*^+^ (1.25 mM) formed in the complex **1**‐catalyzed reduction of O_2_ by Fc* is approximately four times that of the O_2_ concentration (0.30 mM) (Figure S12A). Thus, four‐electron reduction of O_2_ to H_2_O occurs at 20°C.

**FIGURE 6:**
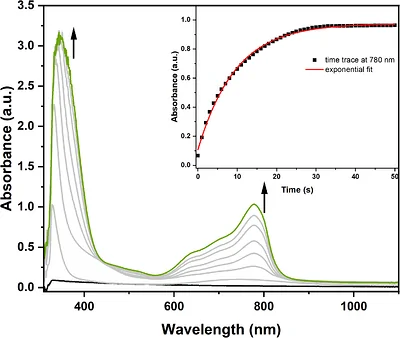
Catalytic ORR using 0.1 mM complex **1**, 2 mM Fc* and 20 mM TFA leading to quantitative formation of Fc*^+^.

The rate of formation of Fc*^+^ obeyed pseudo‐first‐order kinetics under the conditions that [1] ≪ [Fc*] < [TFA] (Figures 6 and S13). The pseudo‐first‐order rate constant (*k*_obs_) increased linearly with increasing concentrations of catalyst **1** (Figure S13A) or TFA (Figure S13B). However, the *k*_obs_ values were independent of the concentrations of Fc* (Figure S13C) and O_2_ (Figure S13D), suggesting that O_2_ binding and e–transfer are fast and do not affect the rate‐determining step. This is inconsistent with the observed reversible binding of O_2_ in the absence of any proton source (Figure S4). Thus, the O_2_ binding to **1**, which is not allowed at 25°C, is significantly facilitated and accelerated under catalytic conditions in the presence of a proton source.

To analyze the subsequent steps of ORR, stoichiometric amounts of Fc* and TFA were added to a preformed solution of **1a** at −90°C. Notably, the addition of Fc* alone did not lead to the decay of **1a**. However, the addition of TFA to **1a** led to an immediate decrease of its optical features (Figures S14 and S15A) with a rate constant of *k*_2_ = 0.487 mM^−1^ s^−1^. When TFA was added in the presence of 1 eq. of Fc*, no significant enhancement of the reaction rate could be observed, suggesting that the step is not going through a proton‐coupled‐electron transfer (PCET) process; rather, a rate‐determining proton transfer (PT) step (Figure S15B). To verify the formation of either H_2_O (4e^−^–4H^+^ product) or H_2_O_2_ (2e^−^–2H^+^ product), product analyses were performed using TiOSO_4_ and iodometric titration methods [18] for the quantification of possible generated H_2_O_2_. No formation of H_2_O_2_ was detected, further suggesting that O_2_ reduction with **1** occurs via a 4e^−^–4H^+^ process to form H_2_O. To exclude the possible formation of H_2_O_2_, which might subsequently get reduced to H_2_O, we examined the formation of Fc*^+^, formed in the complex **1**‐catalyzed reduction of O_2_ by Fc* at −60°C, at which temperature no further reduction of H_2_O_2_ in the presence of **1** or Fc* occurs. It can be observed that, also at low temperature, the concentration of the generated Fc*^+^ (1.24 mM) cation is approximately four times that of the O_2_ concentration (0.3 mM) (Figure S12B). Thus, the catalytic system of **1** displays unusual selectivity towards H_2_O, which is in contrast to previously reported ^E^P systems [33], which are assumed to prefer the 2e^−^–2H^+^ pathway to form H_2_O_2_.

## 3 Conclusion

In summary, we have transformed a Cu(I) complex **2** that is unable to activate O_2_ into a catalyst **1**, which performs 4e^−^/4H^+^ reduction of O_2_ to H_2_O by weakening the ligand field *trans* to the O_2_ binding site by the introduction of an O atom into the N_4_ macrocycle of the 14‐TMC ligand. An *end‐on* peroxodicopper(II) species (**1a**) is formed upon O_2_ binding at low temperature (Scheme 1), which, upon rate‐determining proton transfer followed by fast electron transfer (or HAT from TEMPO‐H), undergoes O─O bond cleavage to form the bis(*μ*‐hydroxo)dicopper(II) species **1b**. Under catalytic turnover conditions, **1b** decays to a Cu(II) species **1c** with the concomitant release of H_2_O. **1c** can be reduced to regenerate **1**. In the absence of any proton source, O_2_ binding is weak and only occurs reversibly at low temperature. Under catalytic conditions, we, therefore, believe that first 14‐TMCO gets protonated in a rate‐determining step, as previously suggested for the corresponding [Cu^I^(12‐TMC)]^+^ complex **3** [33], followed by fast O_2_ binding and O─O bond cleavage, as also supported by our kinetic studies (Figure S8). The accelerated O–O binding can presumably be attributed to the H‐bonding stabilization of the O_2_^2‒^ moiety by the protonated TMCO ligand (Scheme 1) in **1a''** (Scheme 1, left), which may also lead to an efficient O─O bond cleavage to form **1b** in the presence of electron donors. Notably, O_2_ binding can also be initiated in the Cu(I)‐N_4_ platform by forcing the metal ion to move out of the plane formed by the four nitrogen atoms in the contracted [Cu(12‐TMC)]^+^ complex **3** [33]. In this case, a *side‐on* peroxodicopper(II) species **3a** (Scheme 1; right) has been spectroscopically characterized at low temperature [33], which is also proposed to be stabilized by ligand protonation in **3a''**, and its corresponding decay to the 2e^−^/2H^+^ H_2_O_2_ product. Notably, the Cu─O bond is stronger in **3a** relative to **1a**, as evident from ν_Cu‐O_ vibrational modes of 516 cm^−1^ and 544 cm^−1^ in **1a** and **3a**, respectively. Thus, the different ORR selectivity observed in **1** and **3** must be attributed to the differences in the Cu─O bond strengths in the protonated peroxo complexes **1a''** and **3a''** (Scheme 1). Although, the structures of **1a''** and **3a''** are not known, it is intuitively clear that the Cu–O_2_ interaction must be stronger in **1a''** than in **3a''**, presumably because of the weaker Cu─O_TMCO_ bond relative to Cu–N_(12‐TMC)_ *trans* to the bound O_2_^2‒^ ligand, which may prefer a *side‐on* binding mode in **1a''** and an *end‐on* binding mode in **3a''**. This may result in the release of H_2_O_2_ from *end‐on* **3a''** rather than O─O bond cleavage and H_2_O formation from *side‐on* **1a''**. Overall, the present study underlines the critical role of first‐sphere ligand design in controlling the selectivity in O_2_ reduction chemistry. Further, the importance of axial binding modulating the strength of the *trans*‐effect is highlighted, giving a solid foundation for further catalyst design to control O_2_ binding and the 2e^−^–2H^+^ versus 4e^−^–4H^+^ dioxygen reduction processes.

**SCHEME 1:**
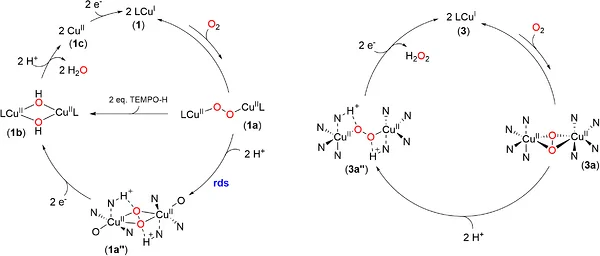
Proposed mechanism of the catalytic O_2_ reduction of **1** (left) and **3** (right) [33] at ambient temperature.

## Conflicts of Interest

The authors declare no conflicts of interest.

## Supporting information




**Supporting File 1**: chem71479‐sup‐0001‐SuppMat.pdf
The authors have included additional spectroscopic and crystallographic data as well as cited references within the Supporting Information [47, 48, 49, 50].

## Data Availability

The data that support the findings of this study are available from the corresponding author upon reasonable request.
