# Supplementary material for: Dioxygen Reduction at a Cu(I) Complex Supported by a Macrocyclic N3O Ligand System
Source: Chemistry. 2026 Aug 14;32(41):e71479. doi: 10.1002/chem.71479 (PMC13729956; doi:10.1002/chem.71479)
Supplement: Supplementary file 1 [file CHEM-32-e71479-s001.pdf]

Supporting Information

Dioxygen Reduction at a Cu(I) Complex Supported by a Macrocyclic N_3O Ligand System

*Maximilian Schütze, Dibya Jyoti Barman, Christian Lorent and Kallol Ray**

Table of Contents

Table of Contents……2

Experimental Procedures……2

Materials and Instrumentations……2

Synthesis Procedures……3

Generation of the intermediates……3

Sample preparation and catalytic studies……3

Results and Discussion……4

Experimental Procedures

Materials and Instrumentations

Chemicals and handling. All chemicals used, including 14-TMC, were purchased from the companies ABCR, SIGMA-ALDRICH, TCI, and BLDpharm and used without further purification unless required. Anhydrous solvents (dimethyl formamide, diethyl ether, dichloromethane and acetone) were purchased from CARL-ROTH GmbH under the tradename ROTIDRY (>99.5%, <50 ppm H_2O). Deuterated solvents were purchased from EURISO-TOP.

Preparation and handling of air or water sensitive compounds were performed under an inert atmosphere using either Schlenk techniques or a GS MEGA M-Line glovebox filled with N_2. Nitrogen and argon of quality 5.0 were used for this purpose and were purchased from AIR LIQUIDE.

Elemental analysis. All elemental analyses were performed by the analytical service of the Institut für Chemie of the Humboldt-Universität zu Berlin. The percentages of Carbon, Hydrogen, Nitrogen and Sulphur were determined using a HEKAtech EURO EA 3000 analyzer. The reported values are the result of an average of two independent measurements.

Nuclear magnetic resonance spectroscopy. All NMR spectra were recorded using a BRUKER AVANCE DPX 300 spectrometer or a BRUKER AVANCE III 500 spectrometer. ^1H and ^{13}C nuclei were recorded in deuterated solvents, and signals (ppm) referenced against residual protic solvent peaks.

Electrospray ionization mass spectrometry. ESI-MS spectra of organic molecules and inorganic complexes in solution were recorded by using an ADVION EXPRESSION CMS spectrometer (in positive ionization mode); acetonitrile was used as eluent. The analysis of the data was carried out with the ADVION DATA EXPRESS Version 6.0.11.3.

Single crystal X-ray diffraction. Data collection was performed at 100 K on a BRUKER D8 VENTURE diffractometer by using Mo Kα = radiation (λ = 0.71073 Å). Multi-scan absorption correction implemented in SADABS[47] was applied to the data. The structure was solved by intrinsic phasing method (SHELXT 2014/5)[48] and refined by full-matrix-least-square procedures based on F^2 with all measured reflections (SHELXL-2018/3)[49] in the graphical user interface (SHELXle)[50] with anisotropic temperature factors for all non-hydrogen atoms. All hydrogen atoms were added geometrically and refined by using a riding model. Crystallographic data are displayed in Table S1-S3 and Table S5-S6. Deposition numbers 2542682 (**1**), 2542679 (**1a**), 2542681 (**1c**), 2542680 (**2**) and 2558833 (**1**-PF_6) contain the supplementary crystallographic data for this paper. These data are provided free of charge by the joint Cambridge Crystallographic Data Centre and Fachinformationszentrum Karlsruhe Access Structures service.[38]

Electron paramagnetic resonance spectroscopy. X-band EPR spectra were collected by a Bruker EMXplus Instrument. All samples were measured as frozen solutions under non-saturating conditions by the use of the liquid helium recirculating cooling system provided by ColdEdge. Data handling and quantification of the signals were performed by comparing the area of the double-integrated experimental signals with a copper(II) standard (1 mM $CuSO_4$ in 9:1 H_2O/glycerol) and the use of OriginPro 2022b software. Typical sample concentration was 1 mM in acetone (temperature: 13 K, modulation amplitude: 5 G, power: 0.016 mW, 41 dB). The experimental spectra were baseline corrected.

Cyclic voltammetry. The CV experiments were performed with a CH-Instrument potentiostat. Acetone for **1** and **2** was used as solvent and tetrabutylammonium hexafluorophosphate ([(n-Bu)$_4$N]PF_6 (TBAPF), 0.1 M) as supporting electrolyte. The CV measurements were performed in a three-electrode setup using a platinum working electrode, a platinum wire counter electrode and a Ag/$AgNO_3$ pseudo reference electrode under nitrogen atmosphere at 20 °C. The potential was referenced against ferrocene which was measured under identical conditions.

Resonance Raman. Resonance Raman spectra were measured in solution state at –95 °C (Bruker cryostat) with 532 nm excitation (frequency-doubled Nd:YAG laser) using a Horiba Jobin-Yvon LabRAM HR800 confocal Raman spectrometer with an Ultra Low Frequency Raman Module (ULF) and Peltier cooled Synapse 1024 x 256 CCD detector. The

concentrations of samples were around 2 mM. Spectra were obtained at a laser power of 1 mW with an accumulation time of about 5 min.

Synthesis Procedures

The synthesis of 14-TMCO were performed according to literature-known procedure and analyzed by ^{1}H-NMR spectroscopy. [34] Metalation reactions were performed under inert conditions.

Synthesis of [Cu^{I}(14-TMCO)](OTf) (1). To a solution of the ligand 14-TMCO (50 mg, 0.21 mmol, 1.05 eq) in acetone (1 mL) was added a solution of Cu^{I}(MeCN)$_4$OTf (74 mg, 0.20 mmol, 1 eq) in acetone (1 mL) and stirred for 30 min at room temperature. The solution was filtered and precipitated in diethyl ether (20 mL). The solvent was decantated and the remaining solid washed another two times with diethyl ether. The solid was dried under reduced pressure and redissolved in acetone (0.5 mL). Diethyl ether diffusion afforded **1** as colorless crystals (114 mg, 0.18 mmol, 90%).

ESI-MS (pos. mode, m/z): [Cu^{I}+L]$^{+}$ calc.: 306.2, exp.: 306.2.

EA (in %) : exp.: C: 36.59, H: 6.19, N: 8.92, S: 7.02, calc.: C: 36.88, H: 6.41, N: 9.21, S: 7.03 for $C_{14}H_{29}CuF_3N_3O_4S$.

Synthesis of [Cu^{I}(14-TMCO)](PF$_6$) (1-PF$_6$**).** To a solution of the ligand 14-TMCO (31 mg, 0.21 mmol, 1.05 eq) in acetone (1 mL) was added a solution of Cu^{I}(MeCN)$_4$PF$_6$ (45 mg, 0.20 mmol, 1 eq) in acetone (1 mL) and stirred for 30 min at room temperature. The solution was filtered and precipitated in diethyl ether (20 mL). The solvent was decantated and the remaining solid washed another two times with diethyl ether. The solid was dried under reduced pressure and redissolved in acetone (0.5 mL). Diethyl ether diffusion afforded **1** as colorless crystals.

ESI-MS (pos. mode, m/z): [Cu^{I}+L]$^{+}$ calc.: 306.2, exp.: 306.3.

Synthesis of [Cu^{II}(14-TMCO)(OTf)](OTf) (1c$_{auth}$). To a solution of the ligand 14-TMCO (50 mg, 0.21 mmol, 1.05 eq) in acetone (1 mL) was added a solution of Cu^{II}(OTf)$_2$ (71 mg, 0.20 mmol, 1 eq) in acetone (1 mL) and stirred for 30 min at room temperature. The solution was filtered and precipitated in diethyl ether (20 mL). The solvent was decantated and the remaining solid washed another two times with diethyl ether. The solid was dried under reduced pressure and redissolved in acetone (0.5 mL). Diethyl ether diffusion afforded **1c**$_{auth}$ as blue crystals (115 mg, 0.19 mmol, 95%).

ESI-MS (pos. mode, m/z): [M+H]$^{+}$ calc.: 319.2, exp.: 319.3.

Synthesis of [Cu^{I}(14-TMC)](OTf) (2). To a solution of the ligand 14-TMC (50 mg, 0.19 mmol, 1.05 eq) in acetone (1 mL) was added a solution of Cu^{I}(MeCN)$_4$OTf (68 mg, 0.18 mmol, 1 eq) in acetone (1 mL) and stirred for 30 min at room temperature. The solution was filtered and precipitated in diethyl ether (20 mL). The solvent was decantated and the remaining solid washed another two times with diethyl ether. The solid was dried under reduces pressure to give **2** as a white powder (75 mg, 0.16 mmol, 89%).

ESI-MS (pos. mode, m/z): [M+H]$^{+}$ calc.: 319.2, exp.: 319.3.

Generation of the intermediates

Formation of intermediate 1a. In a typical experiment, a solution of **1** in acetone was prepared and filled in a cuvette. After cooling down to –90 °C, O_2 was bubbled through the solution for 20 seconds. The immediate formation of the characteristic bands of **1a** could be observed.

Formation of intermediate 1b. In a typical experiment, a solution of **1** in acetone was prepared and filled in a cuvette. After cooling down to –50 °C, O_2 was bubbled through the solution for 10 seconds. The immediate formation of the characteristic bands of **1b** could be observed.

Sample preparation and catalytic studies

General procedure for catalytic ORR. In a typical experiment, **1** was added to an O_2-saturated acetone solution containing 20 mM TFA (total concentration of **1**: 0.05 mM, total volume: 2.1 mL). Subsequently, 0.1 mL of Fc* in acetone

was added (total concentration of Fc*: 1 mM) and the reaction monitored by UV/Vis spectroscopy (λ = 780 nm). For kinetic studies the concentration of **1** (0.1-1 mM), TFA (1-50 mM), O_2 (3-11.5 mM) and Fc* (0.5-2 mM) were varied.

Kinetic studies of ORR at low-temperature. In a typical experiment, a solution of **1** (0.5 mM, cuvette pathlength: 1 cm, cuvette volume: 2 mL) in acetone was prepared and filled in a cuvette. After cooling down to –90 °C, O_2 was bubbled through the solution for 20 seconds. The immediate formation of the characteristic bands of **1a** could be observed. Stochiometric amounts of either TFA or Fc* were added and the reaction followed at the band of 527 nm.

General procedure for H_2O_2 quantification. In a typical experiment, **1** was added to an O_2-saturated acetone solution containing 10 mM TFA (total concentration of **1**: 0.2 mM, total volume: 2 mL). Subsequently, 0.1 mL of Fc* in acetone was added (total concentration of Fc*: 2 mM) and the reaction monitored by UV/Vis spectroscopy (λ = 780 nm).

a) *KI method*: After completion of the reaction (300 s), 0.1 mL of the reaction mixture was added to 1.9 mL of a 0.1 M KI solution in H_2O. No increase of the band at 351 nm was observed indicating no significant formation of H_2O_2. An authentic solution of H_2O_2 was prepared (total concentration of H_2O_2 in KI solution: 0.1 mM) and an increase of the band at 360 nm was observed.

b) *$TiOSO_4$ method*: After completion of the reaction (300 s), 1 mL of the reaction mixture was diluted with 1 mL dist. H_2O and 100 µL of a titanium oxysulfate solution (15 wt% in sulfuric acid) were added. No increase of the characteristic band at 408 nm were observed indicating no formation H_2O_2.

Results and Discussion

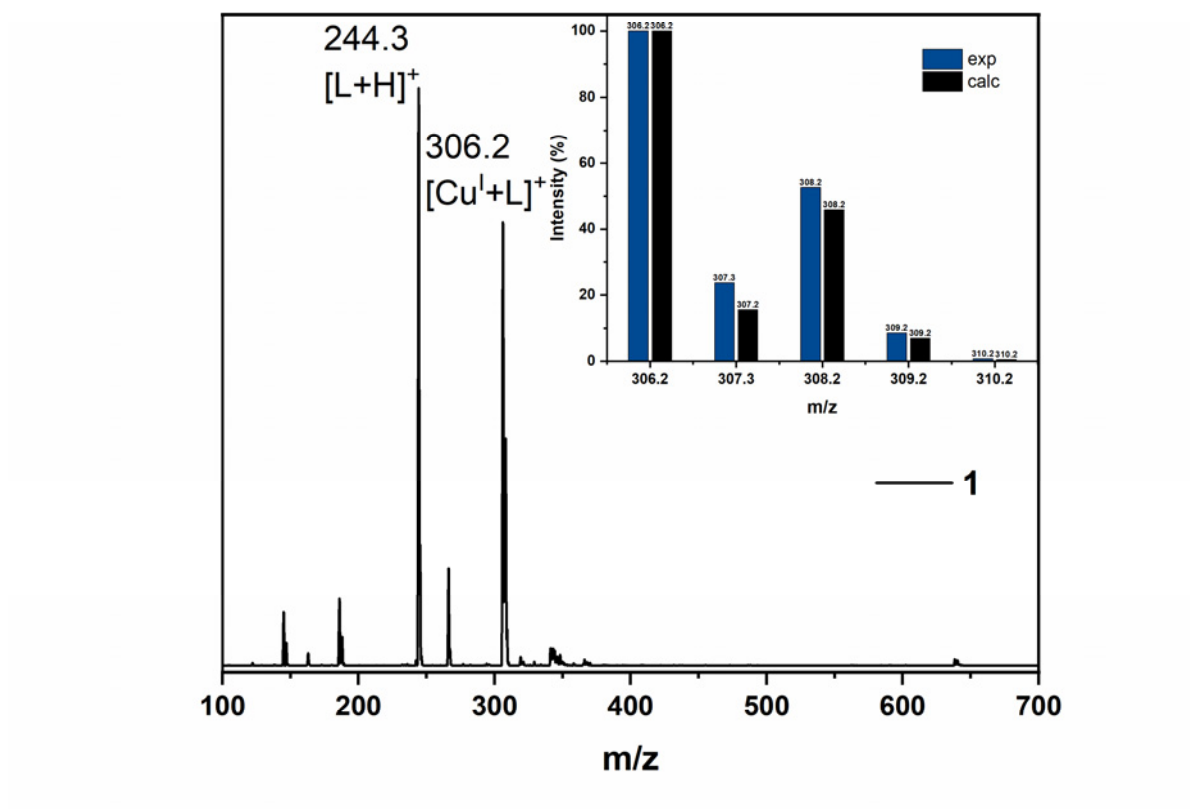

Figure S1. ESI-MS spectrum (positive mode) of **1**; Inset: Comparison of the experimental obtained data (blue) and calculated data (black).

Table S1. Crystallographic data of **1** obtained from sc-XRD measurements.

Chemical sum formula	$C_{14}H_{29}CuF_3N_3O_4S$
Chemical moiety formula	$C_{14}H_{29}CuF_3N_3O_4S$
Molecular weight	456.0 g/mol
Temperature	100(2) K
Wavelength	0.71073 Å
Crystal system	monoclinic
Space group	*P*2/*c*
Unit cell parameters	*a* = 9.2399(3) Å
	b = 11.4359(3) Å
	c = 18.1219(5) Å
	α = 90 °
	β = 90.4470(10) °
	γ = 90 °
Cell volume	V = 1914.82(10) $Å^3$
Z	16
Crystal density	1.582
Absorption coefficient *μ*	1.302
F(000)	952
Crystal size	0.15 x 0.14 x 0.04 mm
Θ_{min} to θ_{max}	2.868 ° to 26.391 °
h, k, l range	$-11 \leq h \leq 11$; $-14 \leq k \leq 14$; $-22 \leq l \leq 22$
Total number of reflections (with I > 2σ(I))	3923
Number of parameters/restraints	238/0
R_1 factor	0.0260
wR_2 factor	0.0604
Goodness of Fit (GooF)	1.036
Deposition number	2542682

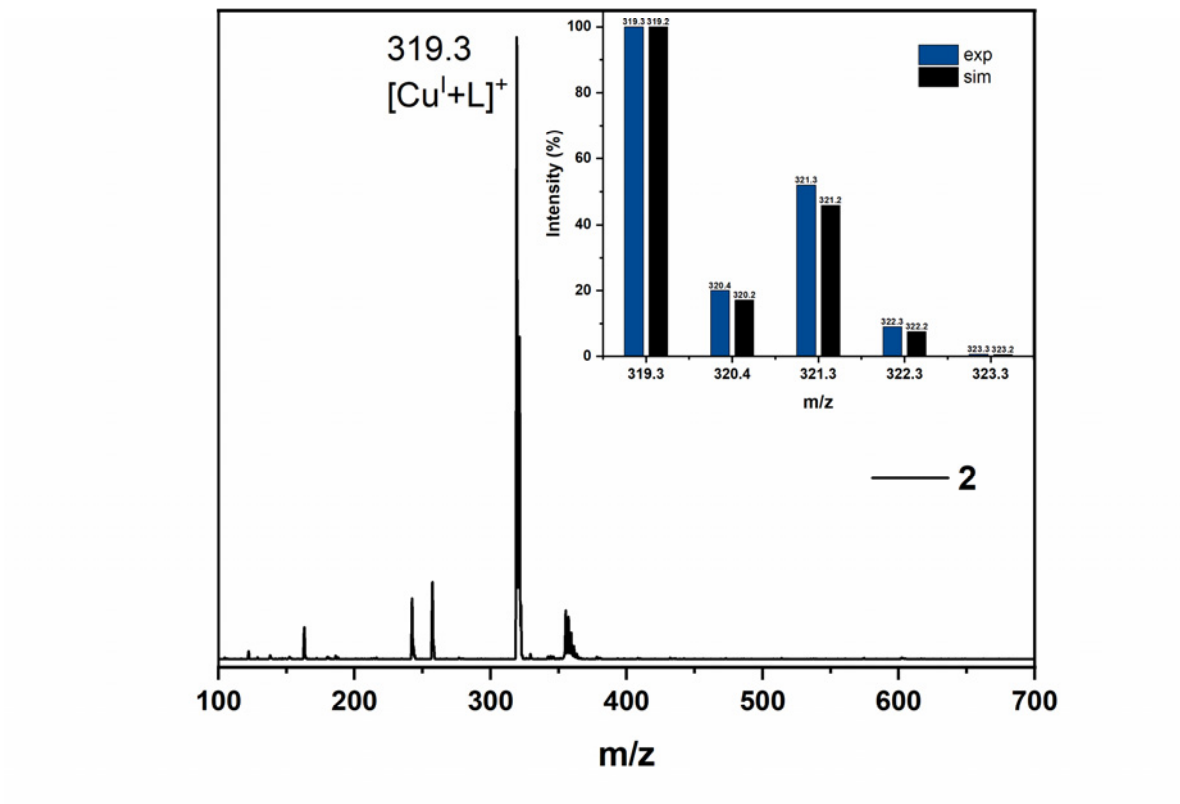

Figure S2. ESI-MS spectrum (positive mode) of **2**; Inset: Comparison of the experimental obtained data (blue) and calculated data (black).

Table S2. Crystallographic data of **2** obtained from sc-XRD measurement.

Chemical sum formula	2 $C_{15}H_{32}CuF_3N_4O_3S$
Chemical moiety formula	2 $C_{15}H_{32}CuF_3N_4O_3S$
Molecular weight	469.0 g/mol
Temperature	100(2) K
Wavelength	0.71073 Å
Crystal system	monoclinic
Space group	*P2*/c
Unit cell parameters	*a* = 11.0089(4) Å
	b = 20.8406(8) Å
	c = 12.5037(4) Å
	α = 90 °
	β = 109.5390(10) °
	γ = 90 °
Cell volume	2703.55(17) $Å^3$
Z	23
Crystal density	1.445
Absorption coefficient *μ*	0.948
F(000)	1240
Crystal size	0.3 x 0.2 x 0.04 mm
Θ_{min} to θ_{max}	2.609 ° to 26.400 °
h, k, l range	-13 ≤ h ≤ 13; -26 ≤ k ≤ 26; -14 ≤ l ≤ 15
Total number of reflections (with I > 2σ(I))	5448
Number of parameters/restraints	323/0
R_1 factor	0.0413
wR_2 factor	0.1056
Goodness of Fit (GooF)	1.074
Deposition number	2542680

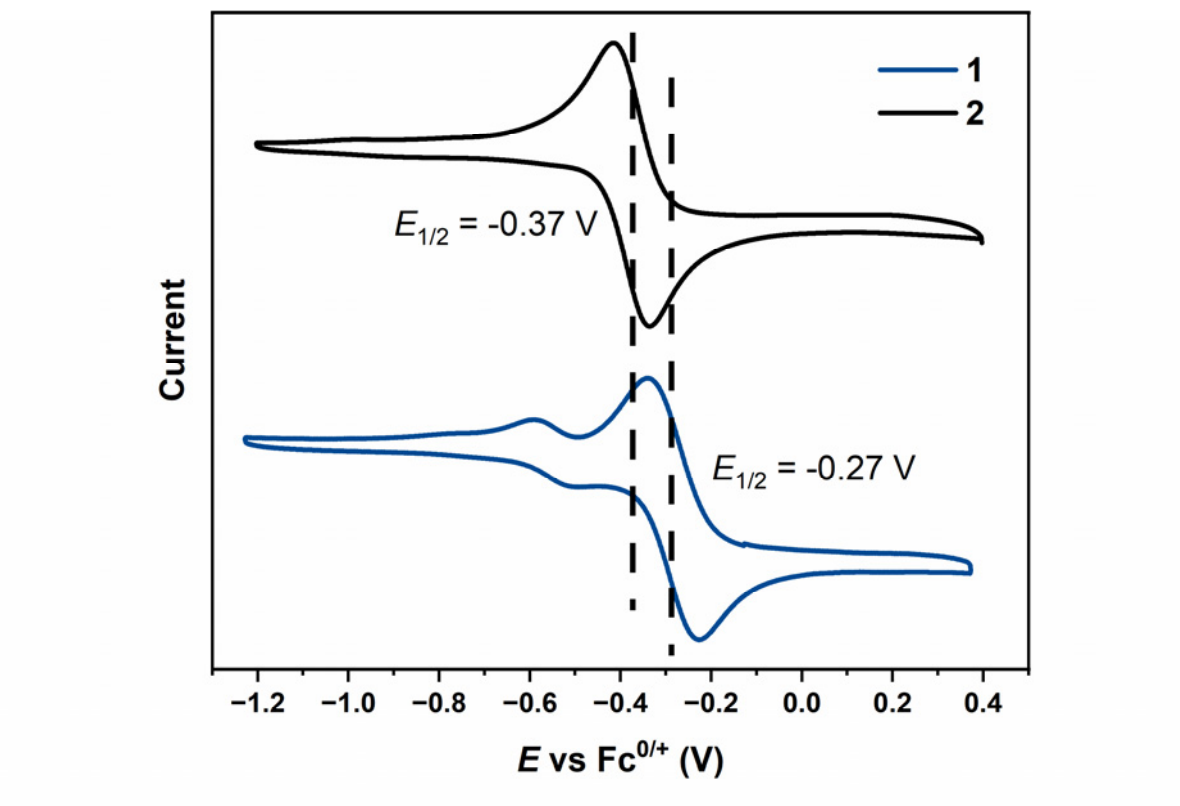

Figure S3. CV spectrum of **1** (blue) and **2** (black) in acetone at 20 °C under anaerobic conditions (concentration: 1 mM, 0.1 M TBAPF, scan rate: 0.1 V/s).

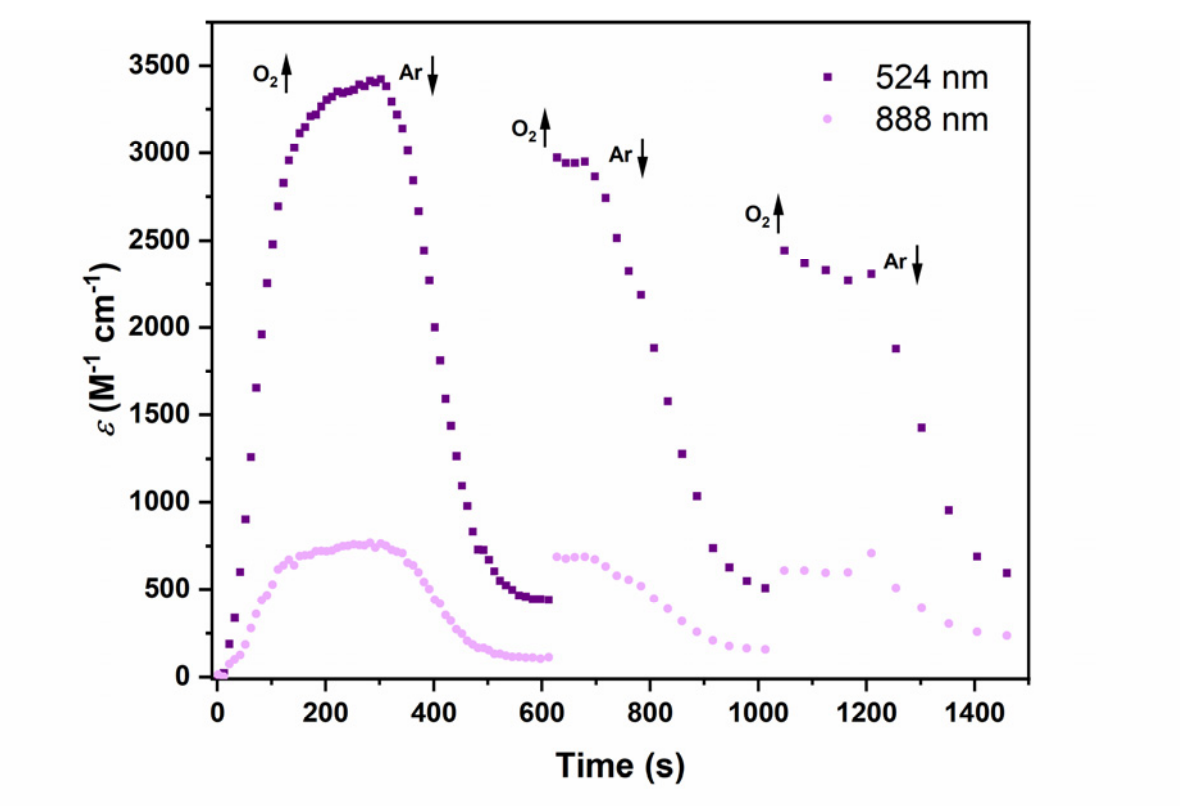

Figure S4. UV/Vis spectral changes (at 527 and 888 nm) upon oxygenation and Ar bubbling through a solution of **1** in acetone at -90 °C.

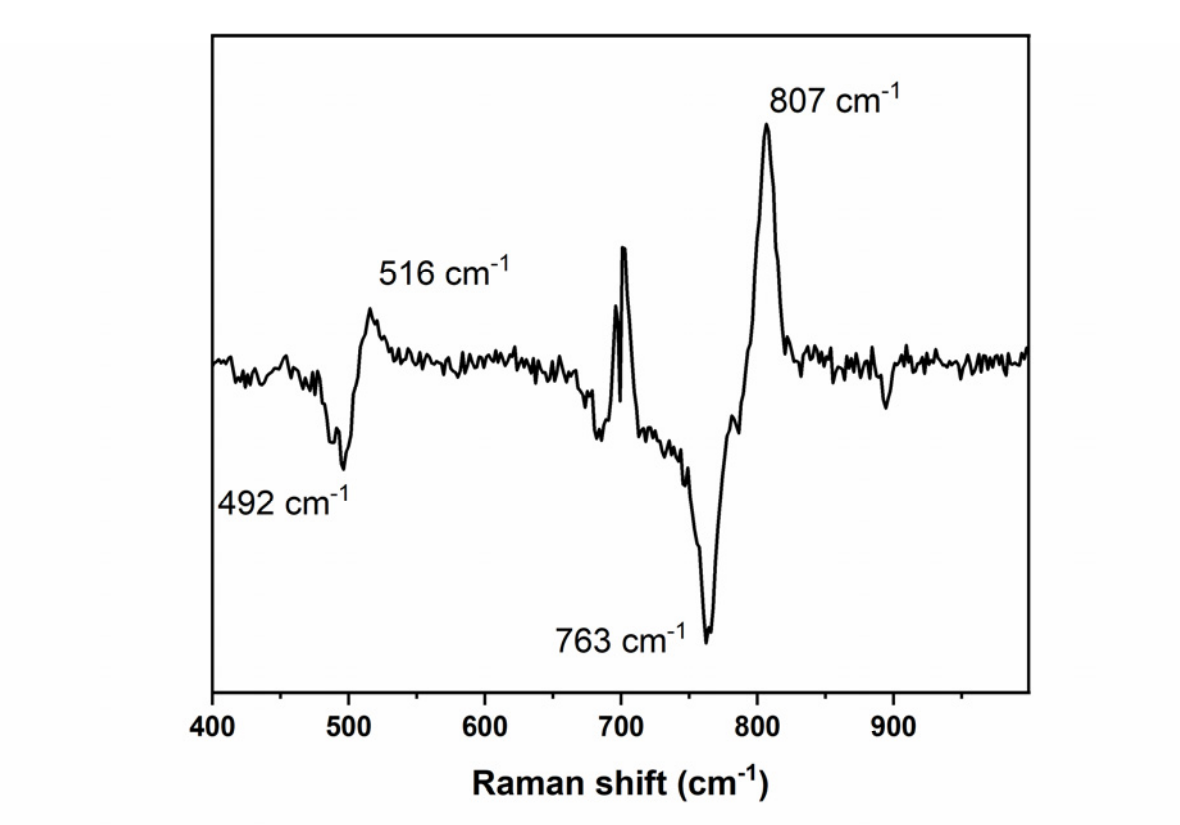

Figure S5. Subtracted rRaman spectrum of **1a** ($^{16}O_2$ and $^{18}O_2$-enriched, temperature: -95 °C, solvent: DCM).

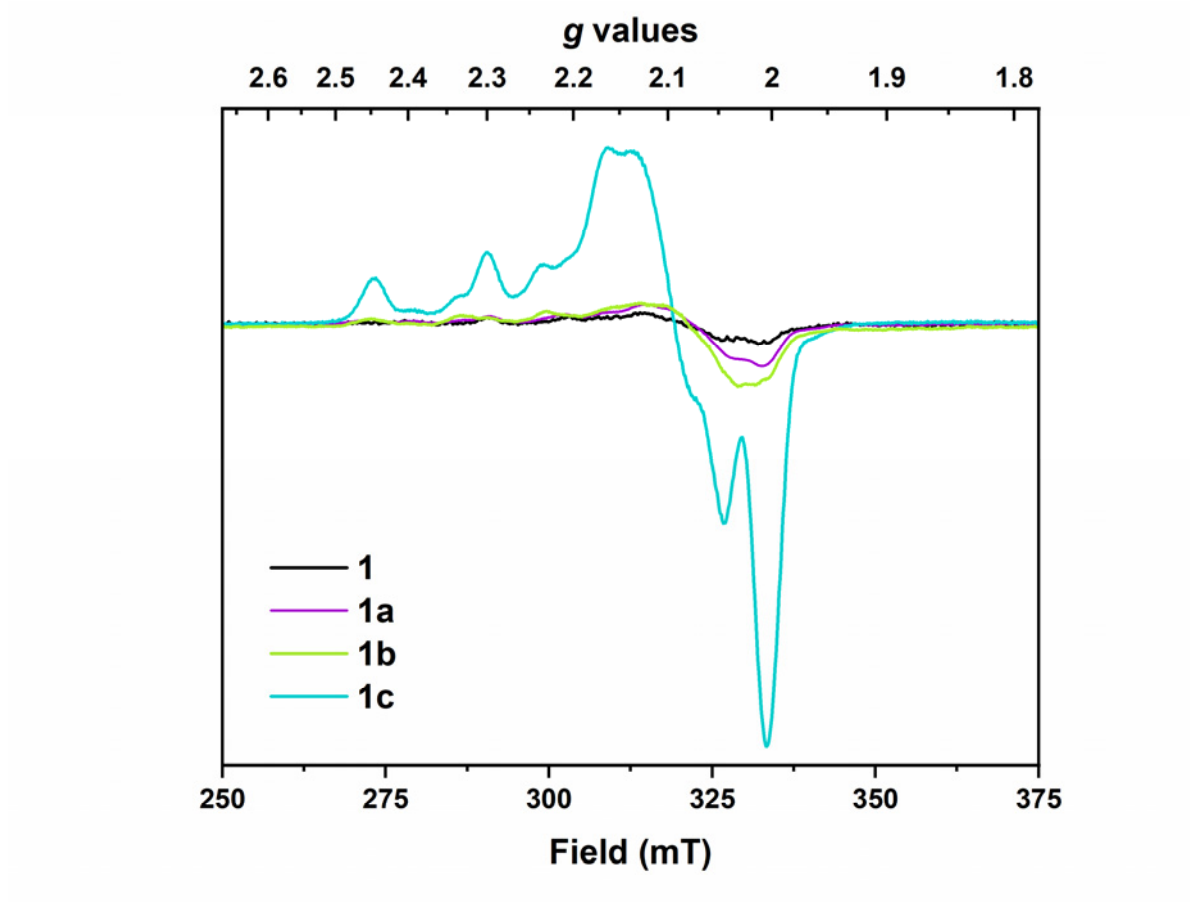

Figure S6. X-band EPR spectrum of **1**, **1a**, **1b** and **1c** in acetone (temperature: 13 K, modulation amplitude: 5 G, power: 0.016 mW, 41 dB). Yield of EPR active species are 3%, 5%, 8% and 40%, respectively, compared to a Cu(II)SO_4 standard under non-saturating conditions.

Table S3. Crystallographic data of **1a** obtained from sc-XRD measurement.

Chemical sum formula	$C_{34}H_{70}Cu_2F_6N_6O_{12}S_2$
Chemical moiety formula	$C_{28}H_{58}Cu_2F_6N_6O_{10}S_2$ x 2 C_3H_6O
Molecular weight	1060.2 g/mol
Temperature	100(2) K
Wavelength	0.71073 Å
Crystal system	monoclinic
Space group	*P2*/c
Unit cell parameters	*a* = 11.0089(4) Å
	b = 20.8406(8) Å
	c = 12.5037(4) Å
	α = 90 °
	β = 109.5390(10) °
	γ = 90 °
Cell volume	2703.55(17) Å^3
Z	23
Crystal density	1.445
Absorption coefficient *μ*	0.948
F(000)	1240
Crystal size	0.3 x 0.2 x 0.04 mm
Θ_{min} to θ_{max}	2.610 ° to 26.399 °
h, k, l range	-13 ≤ h ≤ 13; -26 ≤ k ≤ 26; -14 ≤ l ≤ 15
Total number of reflections (with I > 2σ(I))	5448
Number of parameters/restraints	323/0
R_1 factor	0.0413
wR_2 factor	0.1056
Goodness of Fit (GooF)	1.074
Deposition number	2542679

Table S4. Comparison of bond lengths (in Å) of **1** with reported *end-on* and *side-on* peroxodicopper(II) species obtained from sc-XRD measurements or DFT calculations.[33, 35-37]

bond	1a	$[Cu^{II}_2(tmpa)_2(\mu\text{-}1,2\text{-}O_2)]^{2+}$ [36]	$[Cu^{II}_2(L^{H,Bn})_2(\mu\text{-}1,2\text{-}O_2)]^{2+}$ [37]	$[Cu^{II}_2(12\text{-}TMC)_2(\mu\text{-}1,1\text{-}O_2)]^{2+}$ [33](a)	$[Cu^{II}_2(tet\ b)_2(\mu\text{-}1,2\text{-}O_2)]^{2+}$ [35]
Cu···Cu	4.6817(5)	4.359(1)	4.476(2)	3.51	4.5667
$Cu–O_{peroxo}$	1.910(2)	1.852(5)	1.868(3)	1.95	1.932(3)
O–O	1.444(2)	1.432(6)	1.450(2)	1.47	1.495(4)
$Cu–N_{lig}$	2.204(2), 2.057(2), 2.045(2)	2.104(6)	2.149(3), 2.083(3), 2.142(3), 2.104(4)	2.32	2.026(3), 2.073(4), 2.108(3), 2.249(4)
$Cu–O_{TMCO}$	2.151(2)				

(a) Bond lengths of DFT calculated structure.

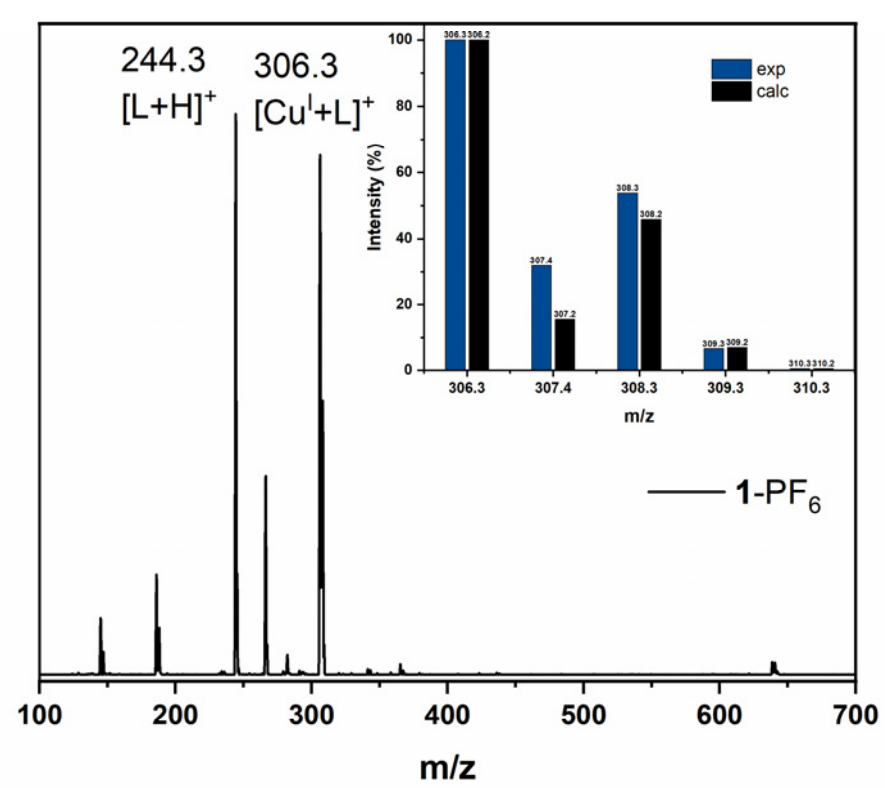

Figure S7. ESI-MS spectrum (positive mode) of **1**-PF_6; Inset: Comparison of the experimental obtained data (blue) and calculated data (black).

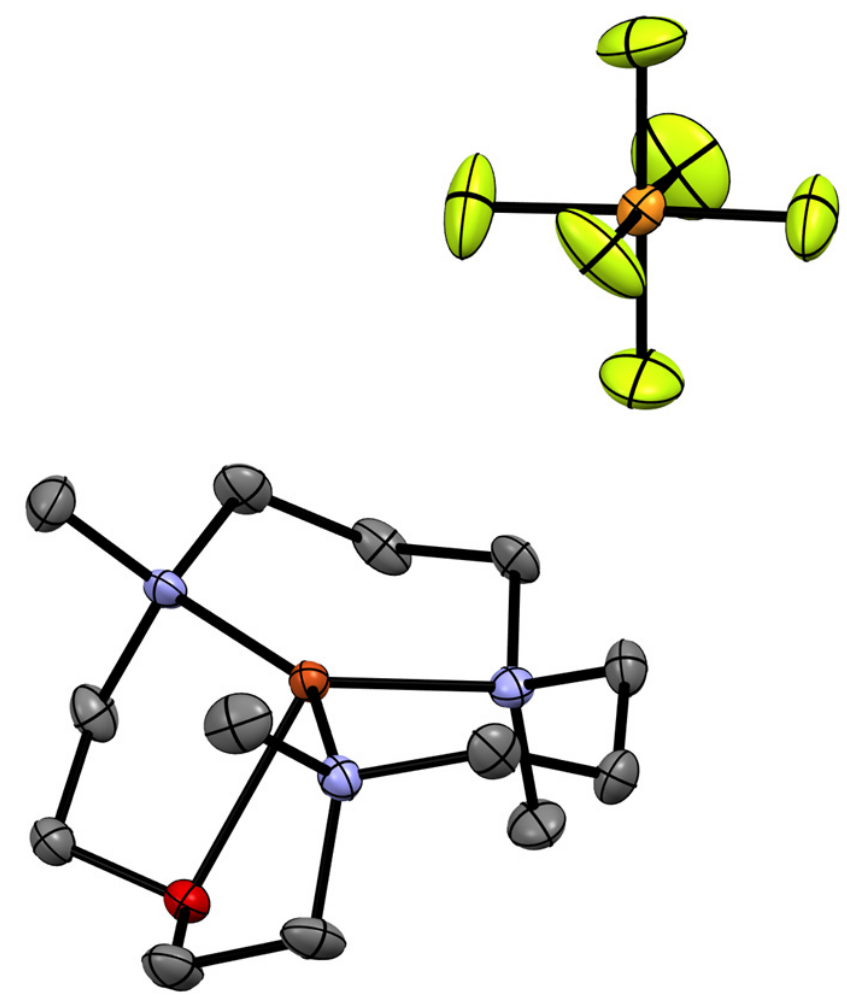

Figure S8. Molecular structure of **1**-PF_6 obtained by sc-XRD measurements in ORTEP representation with thermal ellipsoids at 50% probability level. H-atoms are omitted for clarity. (Color code: C: grey, N: blue, O: red, Cu: orange, P: light orange, F: green).[38]

Table S5. Crystallographic data of **1**-PF_6 obtained from sc-XRD measurement.

Chemical sum formula	$C_{14}H_{33}CuF_6N_3OP$
Chemical moiety formula	$C_{14}H_{33}CuF_6N_3OP$
Molecular weight	467.9 g/mol
Temperature	100(2) K
Wavelength	0.71073 Å
Crystal system	orthorhombic
Space group	*P2*/1
Unit cell parameters	*a* = 8.6297(4) Å
	b = 13.4331(10) Å
	c = 16.3242(12) Å
	α = 90 °
	β = 90 °
	γ = 90 °
Cell volume	1892.4(2) $Å^3$
Z	16
Crystal density	1.586
Absorption coefficient *μ*	1.302
F(000)	936
Crystal size	0.08 x 0.05 x 0.02 mm
Θ_{min} to θ_{max}	2.560 ° to 23.634 °
h, k, l range	-10 ≤ h ≤ 8; -16 ≤ k ≤ 16; -20 ≤ l ≤ 20
Total number of reflections (with I > 2σ(I))	3882
Number of parameters/restraints	229/0
R_1 factor	0.0431
wR_2 factor	0.0925
Goodness of Fit (GooF)	1.059
Deposition number	2558833

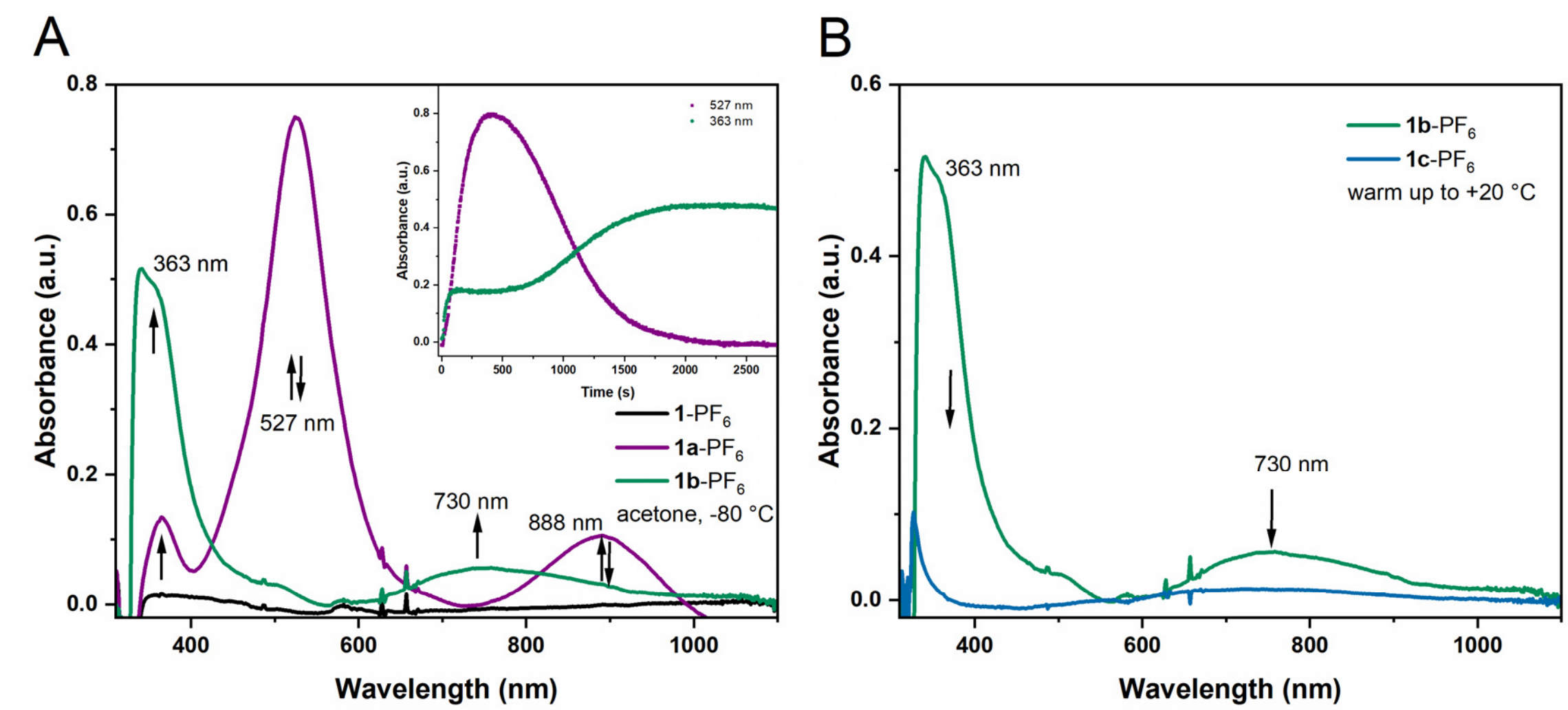

Figure S9. A) UV/Vis spectral changes associated with the reaction of **1**-PF_6 with O_2 (temperature: -80 ºC, solvent: acetone); B) UV/Vis spectral changes upon warming up to 20 °C.

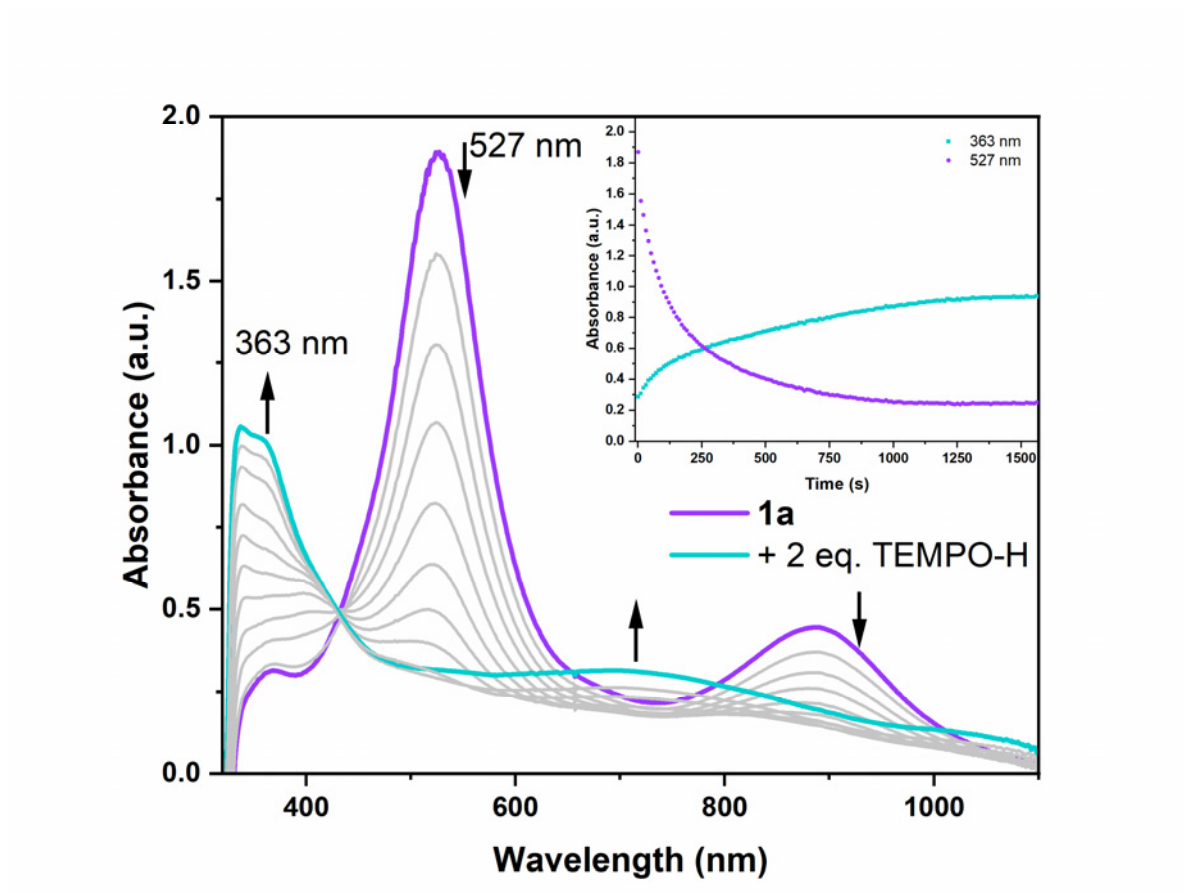

Figure S10. UV/Vis spectral changes associated with the reaction of **1a** with 2 eq. of TEMPO-H (concentration: 0.5 mM, temperature: -90 ºC, solvent: acetone) to form **1b**.

Table S6. Crystallographic data of **1c** obtained from sc-XRD measurement.

Chemical sum formula	$C_{15}H_{29}CuF_6N_3O_7S_2$
Chemical moiety formula	$C_{15}H_{29}CuF_6N_3O_7S_2$
Molecular weight	605.1 g/mol
Temperature	100(2) K
Wavelength	0.71073 Å
Crystal system	monoclinic
Space group	*P2*/n
Unit cell parameters	*a* = 11.0321(6) Å
	b = 15.9320(8) Å
	c = 13.2870(6) Å
	α = 90 °
	β = 94.988(2) °
	γ = 90 °
Cell volume	2326.5(2) $Å^3$
Z	20
Crystal density	1.727
Absorption coefficient *μ*	1.209
F(000)	1244
Crystal size	0.270 x 0.120 x 0.070 mm
Θ_{min} to θ_{max}	2.56 ° to 34.57 °
h, k, l range	-17 ≤ h ≤ 17; -25 ≤ k ≤ 25; -21 ≤ l ≤ 20
Total number of reflections (with I > 2σ(I))	9978
Number of parameters/restraints	310/0
R_1 factor	0.0303
wR_2 factor	0.0721
Goodness of Fit (GooF)	1.074
Deposition number	2542681

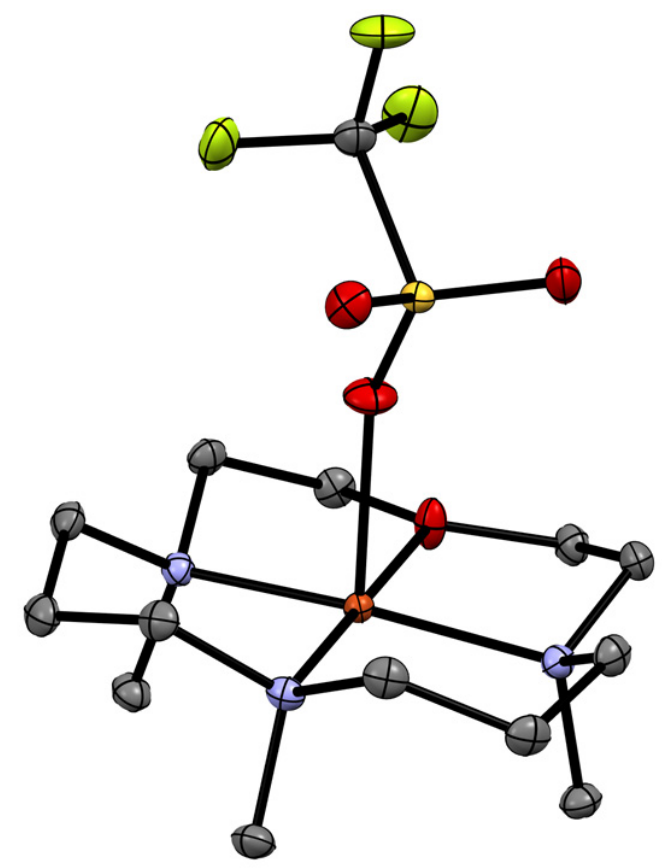

Figure S11. Molecular structure of **1c** obtained by sc-XRD measurements in ORTEP representation with thermal ellipsoids at 50% probability level. H-atoms and one OTf anion are omitted for clarity. (Color code: C: grey, N: blue, O: red, Cu: orange, F: green, S: yellow).

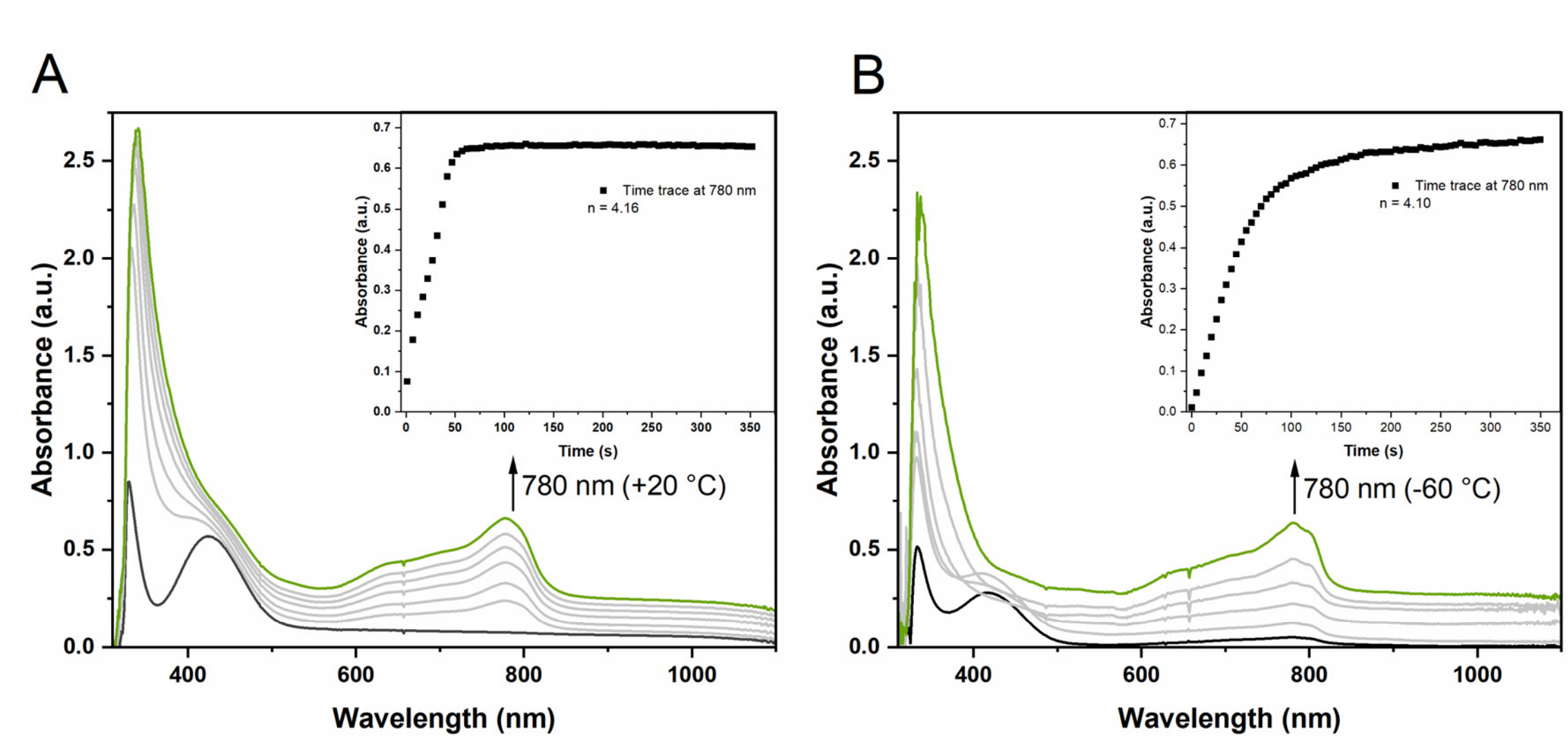

Figure S12. Catalytic ORR with 0.2 mM of complex **1**, 2 MM of Fc*, 10 mM of TFA and limited amount of O_2 (0.3 mM) at A) 20 °C and B) -60 °C for determination of the total converted amount of Fc* (with $\varepsilon_{780\ nm}$ = 520 M^{-1} cm^{-1}, cuvette pathlength d = 1 cm); Inset: Time trace at 780 nm showing the formation of around 1.25 (1.24) mM Fc* which is around four times higher than the amount of O_2.

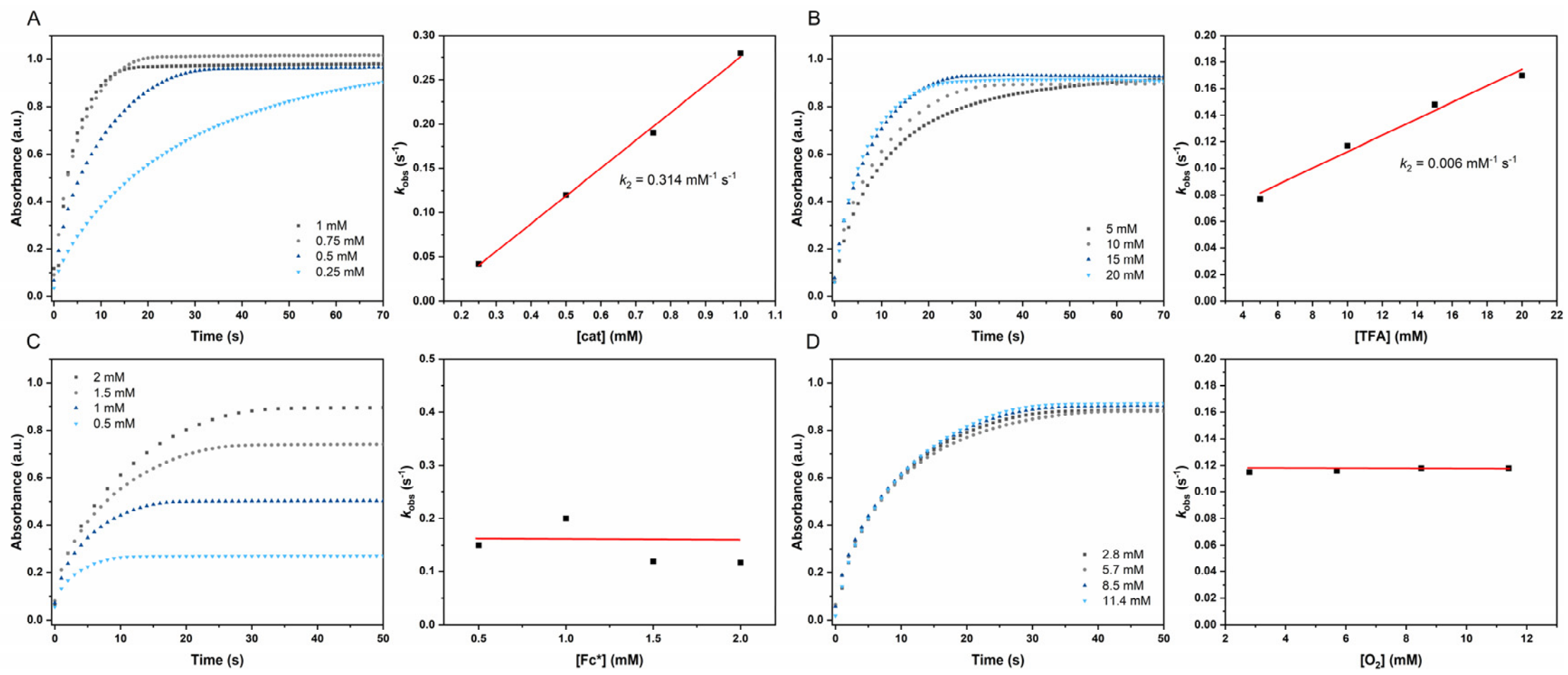

Figure S13. Determination of k_2 values of concentration dependent studies on ORR at room temperature. A) Concentration of catalyst against k_{obs} values, B) Concentration of TFA against k_{obs} values, C) Concentration of Fc* against k_{obs} values and D) Concentration of O_2 against k_{obs} values.

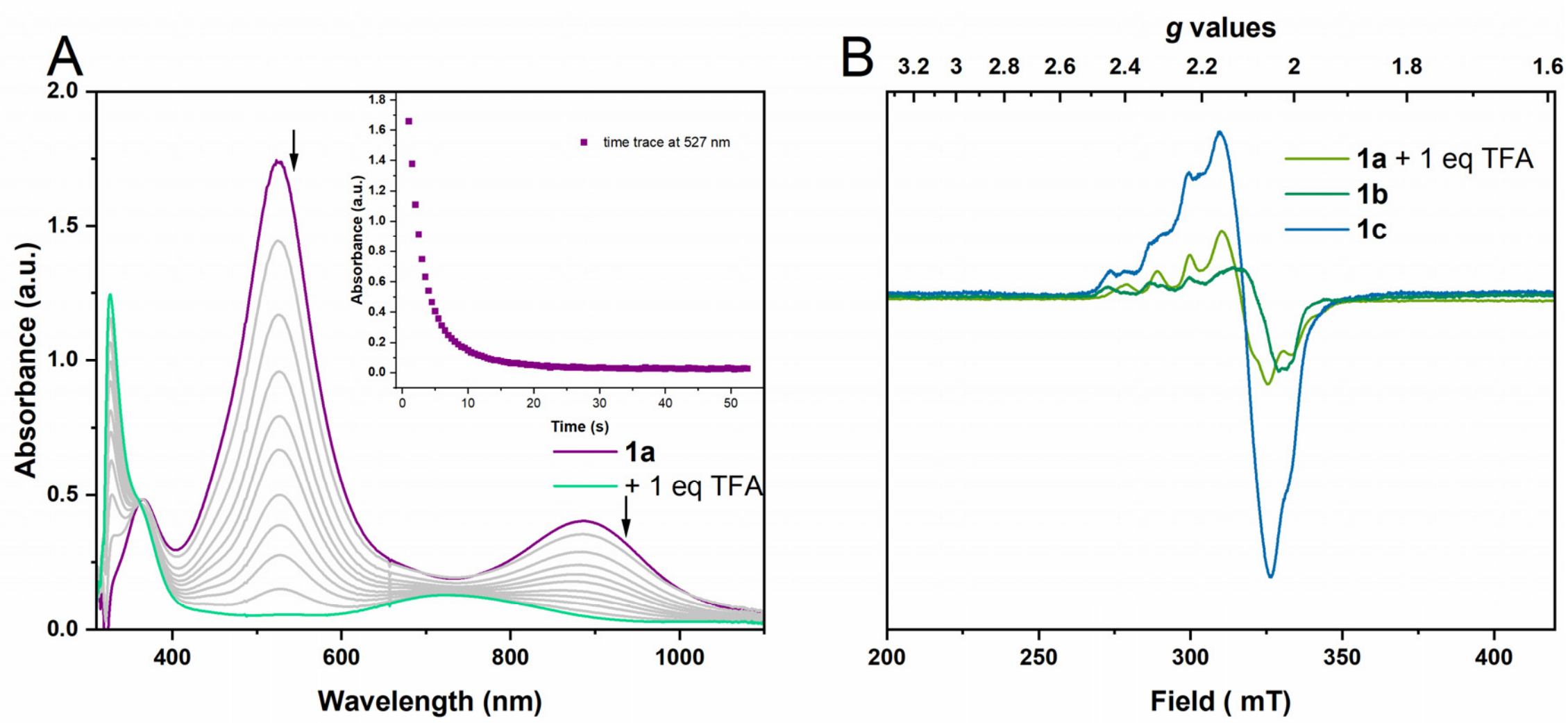

Figure S14. A) UV/Vis spectrum of the reaction of **1a** with 1 eq TFA; Inset: Time trace at 527 nm. B) X-band EPR spectrum of the reaction of **1a** with 1 eq TFA (light-green, Yield: 12%) with comparison of **1b** (dark-green) and **1c** (blue).

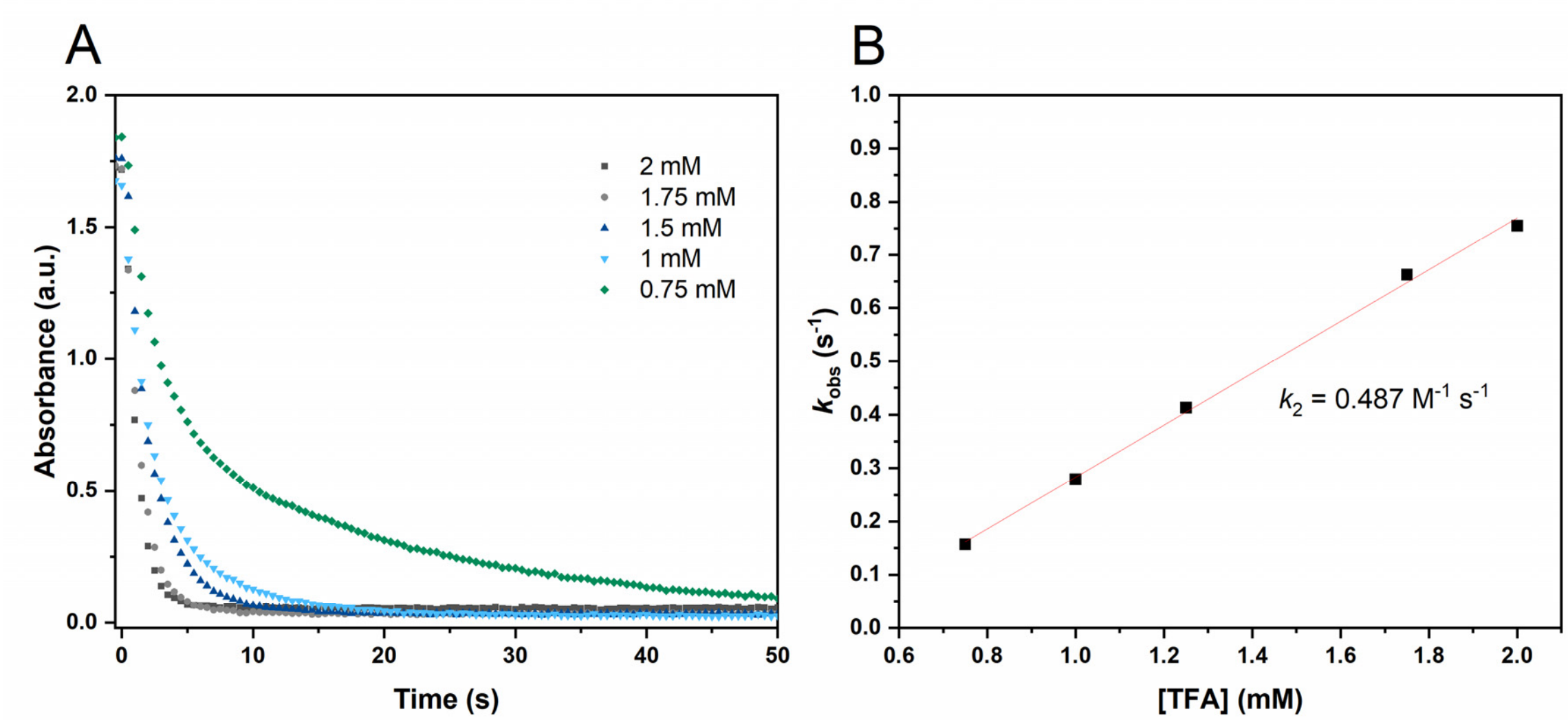

Figure S15. A) UV/Vis spectrum of the reaction of **1a** with different equivalents of TFA. B) Determination of k_2 values of concentration dependent studies on the reaction of **1a** with TFA (concentration of TFA against k_{obs} values).
